# Subcellular Localization of Matrin 3 Containing Mutations Associated with ALS and Distal Myopathy

**DOI:** 10.1371/journal.pone.0142144

**Published:** 2015-11-03

**Authors:** M. Carolina Gallego-Iradi, Alexis M. Clare, Hilda H. Brown, Christopher Janus, Jada Lewis, David R. Borchelt

**Affiliations:** 1 Department of Neuroscience, Center for Translational Research in Neurodegenerative Disease, McKnight Brain Institute, University of Florida, Gainesville, Florida, United States of America; 2 Santa Fe HealthCare Alzheimer’s Disease Research Center, University of Florida, Gainesville, Florida, United States of America; Children's Hospital of Pittsburgh, University of Pittsburgh Medical Center, UNITED STATES

## Abstract

**Background:**

Mutations in *Matrin 3* [MATR3], an RNA- and DNA-binding protein normally localized to the nucleus, have been linked to amyotrophic lateral sclerosis (ALS) and distal myopathies. In the present study, we have used transient transfection of cultured cell lines to examine the impact of different disease-causing mutations on the localization of Matrin 3 within cells.

**Results:**

Using CHO and human H4 neuroglioma cell models, we find that ALS/myopathy mutations do not produce profound changes in the localization of the protein. Although we did observe variable levels of Matrin 3 in the cytoplasm either by immunostaining or visualization of fluorescently-tagged protein, the majority of cells expressing either wild-type (WT) or mutant Matrin 3 showed nuclear localization of the protein. When cytoplasmic immunostaining, or fusion protein fluorescence, was seen in the cytoplasm, the stronger intensity of staining or fluorescence was usually evident in the nucleus. In ~80% of cells treated with sodium arsenite (Ars) to induce cytoplasmic stress granules, the nuclear localization of WT and F115C mutant Matrin 3 was not disturbed. Notably, over-expression of mutant Matrin 3 did not induce the formation of obvious large inclusion-like structures in either the cytoplasm or nucleus.

**Conclusions:**

Our findings indicate that mutations in Matrin 3 that are associated with ALS and myopathy do not dramatically alter the normal localization of the protein or readily induce inclusion formation.

## Introduction

Amyotrophic Lateral Sclerosis (ALS) is characterized by degeneration of both upper and lower motor neurons with usually no more than 5 years survival after symptom onset [1]. More than 10% of cases with ALS develop Fronto Temporal Dementia (FTD) [2]. Although most cases of ALS have no known cause, a subset of ALS is inherited as familial disease. One of the newest gene mutations to be associated with familial ALS (fALS) is *Matrin 3*, which encodes a 125 kDa nuclear matrix protein [3]. Multiple mutations in Matrin 3 have been associated with ALS and paralytic disease caused by myopathy. Late-onset autosomal-dominant ALS associated with a mutation of serine 85 to cysteine (S85C) in Matrin 3 was found in patients with slowly progressing disease [3]. This same mutation was reported as the cause of autosomal dominant, distal, asymmetrical myopathy with vocal cord paralysis in North American families [4–6] and a Bulgarian family with similar disorder [5]. A second mutation of phenylalanine 115 to cysteine (F115C) was identified in affected members of a family of European ancestry with ALS and individuals with no known family history of the disorder [3,7]. The duration of disease associated with the F115C mutations was approximately 5 years. A mutation of threonine 622 to alanine (T622A) was found in a family with Sardinian origin, causing a typical, rapidly familial progressive ALS phenotype [3]. A mutation of proline 154 to serine (P154S) was found in one case of sporadic ALS [3]. Antibodies to Matrin 3 reveal a fibro-granular pattern of nuclear staining [8], and initial reports of Matrin 3 localization in patients harboring ALS mutations indicated a largely nuclear localization with occasional immunostaining in the cytoplasm [3]. It has also been reported that Matrin 3 interacts with the RNA/DNA binding protein TDP-43 [3], which accumulates in cytoplasmic inclusions in ALS patients [9]. Because mutations in other RNA/DNA binding proteins, FUS/TLS and TDP-43, involved in ALS cause mislocalization to the cytoplasm (for review see [10]), we undertook a study to assess the impact of different ALS mutations on the localization of Matrin 3 within cells.

In the present study, we used both immunostaining of transfected cells and expression of Matrin 3 fused to yellow fluorescent protein (YFP), to determine whether mutations in Matrin 3 that cause ALS/myopathy changes the subcellular localization of the protein. Additionally, we have analyzed the localization of Matrin 3 in cells containing stress granules. In response to certain types of stress, eukaryotic cells produce cytoplasmic stress granules, which form to sequester cytoplasmic mRNAs including those encoding pro-apoptotic proteins [11,12]. These granules also contain several RNA-binding proteins, such TDP-43 [13,14] and G3BP1 (Ras-GTPase-activating protein SH3 domain-binding 1) linking signal transduction to RNA metabolism (for review see [15]). Fluorescent fusion proteins of G3BP1 have been used a marker for stress granules [16–18], and we have used co-transfection of G3BP1-mCherry with Matrin 3 fused to yellow fluorescent protein (YFP) as a means to determine whether Matrin 3 may be translocated to stress granules. Our data indicate that Matrin 3 encoding disease-associated mutations does not differ dramatically in its localization from WT protein. Although the protein can be found in the cytoplasm in a subset of cells, in the majority of cells expressing WT and mutant variants, the protein is localized in the nucleus, even under stress conditions.

## Methods

### Cloning of vector plasmids

*MATR3* cDNA was obtained from Thermo Scientific (catalog number MHS6278-202757255; Waltham, MA, USA). PCR was performed using this cDNA as template with engineered primers that enabled cloning into a version the pEF-BOS vector [19] we have previous modified to encode a *Sal 1* site downstream of the transcription start site [20]. cDNA for *Matrin 3* was inserted into the vector using the InFusion HD kit from Clontech (catalog number 639649, Mountain View, CA). The ALS/myopathy mutations (S85C, F115C, P154S, and T622A) were made by PCR site directed mutagenesis of the pEF.Bos vector for WT *Matrin 3*, using the Quick-change kit (Invitrogen, Carlsbad, CA). All modified cDNAs were verified by DNA sequencing.

Fusion cDNAs for *Matrin 3* and yellow fluorescent protein (YFP) were generated by amplifying *Matrin 3* cDNAs (wild-type and mutants) by PCR, using the pEF-BOS vectors as templates, with forward and reverse primers that enabled insertion into a pEF-BOS-YFP vector, using the Infusion HD kit. *GTPase Activating Protein (SH3 Domain) Binding Protein 1* (*G3BP1)* cDNA fused to mCherry in a pEZ-M56 vector was obtained commercially from GeneCopoeia (Cat N_o_ EX-P0037-M56, Rockville, MD, USA). G3BP1-mCherry was used as a marker for stress granules [16–18].

### Cell lines, transfection and immunohistochemistry

CHO cells (ATCC, Manassas, VA) were cultured in F-12K Medium from Thermo Scientific (catalog number 21127–022) plus 10% fetal bovine serum and 2 mM glutamine. H4 cells (ATCC) were cultured in Dulbecco’s Modified Eagle’s Medium from Corning (catalog number 10-080-CV, Corning, NY, USA) supplemented with 10% fetal bovine serum and 2mM glutamine and both were cultured at 37°C and 5% CO_2_.

Cell transfection was performed on coverslips previously coated with 0.5 mg/ml poly-L-lysine in PBS. Cells were seeded in 6-well plates at 25x10^4^ densities (containing coated coverslips) in log-phase growth 24 h previous to transfection. Lipofectamine 2000 and LTX (Invitrogen, Carlsbad, CA) were used for transient transfection following manufacturer protocols. In experiments in which only Matrin 3 was expressed, a total of 1ug/well of *Matrin 3* vector DNA was used. For co-transfections of *Matrin 3*-YFP with the *G3BP1*-mCherry reporter, we used 1.5 μg/well of *Matrin 3* vector with 0.5 μg/well of *G3BP1* vector. Each transfection was repeated at least three times and each transfection was performed in duplicate. The analysis of Matrin 3 localization was performed by an investigator that was blind to cell genotype.

### Immunostaining

Cells grown on coverslips were rinsed with phosphate-buffered saline (PBS), fixed in 4% paraformaldehyde in PBS for 15 min at 25°C, and then permeabilized by 5 min of treatment in −20°C methanol. After incubation in blocking buffer (20% normal goat serum in PBS) for 1 h at 25°C, the cells were exposed to α-Matrin primary antibody at a 1:200 dilution (ab 151714, Abcam, Cambridge, MA) overnight at 4°C. After three 5-min washes in PBS, the cells were then incubated for 1h with secondary antibody, Alexafluor goat anti-rabbit 568, before three washes in PBS (5-min each). A final incubation with 4’,6-diamidino-2-phenylindole, dihydrochloride, stock 14.3 mM (DAPI) (Invitrogen, Carlsbad, CA) for 10’ was followed by one 5-min wash in PBS. Cells were then mounted with Vectashield (Vectorlabs, Burlingame, CA, USA) mounting media for fluorescence and viewed using an Olympus BX60 microscope. Images were obtained using a digital camera Olympus DP71 and Micro DP-BSW controller software. The analysis of Matrin 3 localization was performed by an investigator that was blind to cell genotype.

### Sodium arsenite treatment

Cells were exposed to sodium (meta) arsenite ≥90% (Sigma-Aldrich, St. Louis, MO, USA) at a final concentration of 100 μM diluted in cell culture media. At 24 h post-transfection, cells were washed in PBS and the media was replaced with media containing sodium arsenite for 45 minutes at 37°C to activate a stress response. The cells were then washed twice with phosphate-buffered saline (PBS), fixed in 4% paraformaldehyde in PBS for 15 min at 25°C, rinsed three times with PBS (10 minutes each wash), treated with 4’,6-diamidino-2-phenylindole dihydrochloride stock 14.3 mM (DAPI) (Invitrogen) for 10 minutes, followed by a final 5-min wash in PBS. Cells, fixed to the coverslips, were then mounted with Vectashield (Vectorlabs) mounting media for fluorescence and were imaged 24–36 h after transfection for co-localization using an Olympus BX60 microscope. Images were obtained using a digital camera Olympus DP71 and Micro DP-BSW controller software.

### Statistical methods

Our analysis of localization presents a large data set of frequencies of localization of wild type and mutant Matrin 3 in nuclear or nuclear/cytoplasmic compartments. Since the data are represented by discrete category measures, and on a nominal scale the data did not meet the assumption of parametric statistics, a χ^2^ test of independence was used to test the homogeneity between the groups. Phi (ϕ) correlation coefficient was used as a measure of association or effect size for the variables. For the analysis, we used the frequencies of nuclear only and nuclear/cytoplasmic localization of tagged and untagged Matrin 3. Both categories of nuclear and nuclear/cytoplasmic localization were mutually exclusive, which guaranteed the independence of the scores. However, since the global analysis of multiple mutants and of WT control groups could mask subtle differences between cells expressing WT or mutant Matrin 3 individually, we also performed post-hoc pair-wise comparison of frequencies between each mutant and the WT protein localization. In the case of 2 × 2 contingency tables when sample sizes were small, Fisher Exact Probability test was employed. To control for the increase in familywise error rate due to the multiple comparisons, we adopted more conservative critical level of α = 0.01. All frequencies were weighted before the analyses to adjust the collected data to represent the population estimates.

## Results

**Fig 1:**
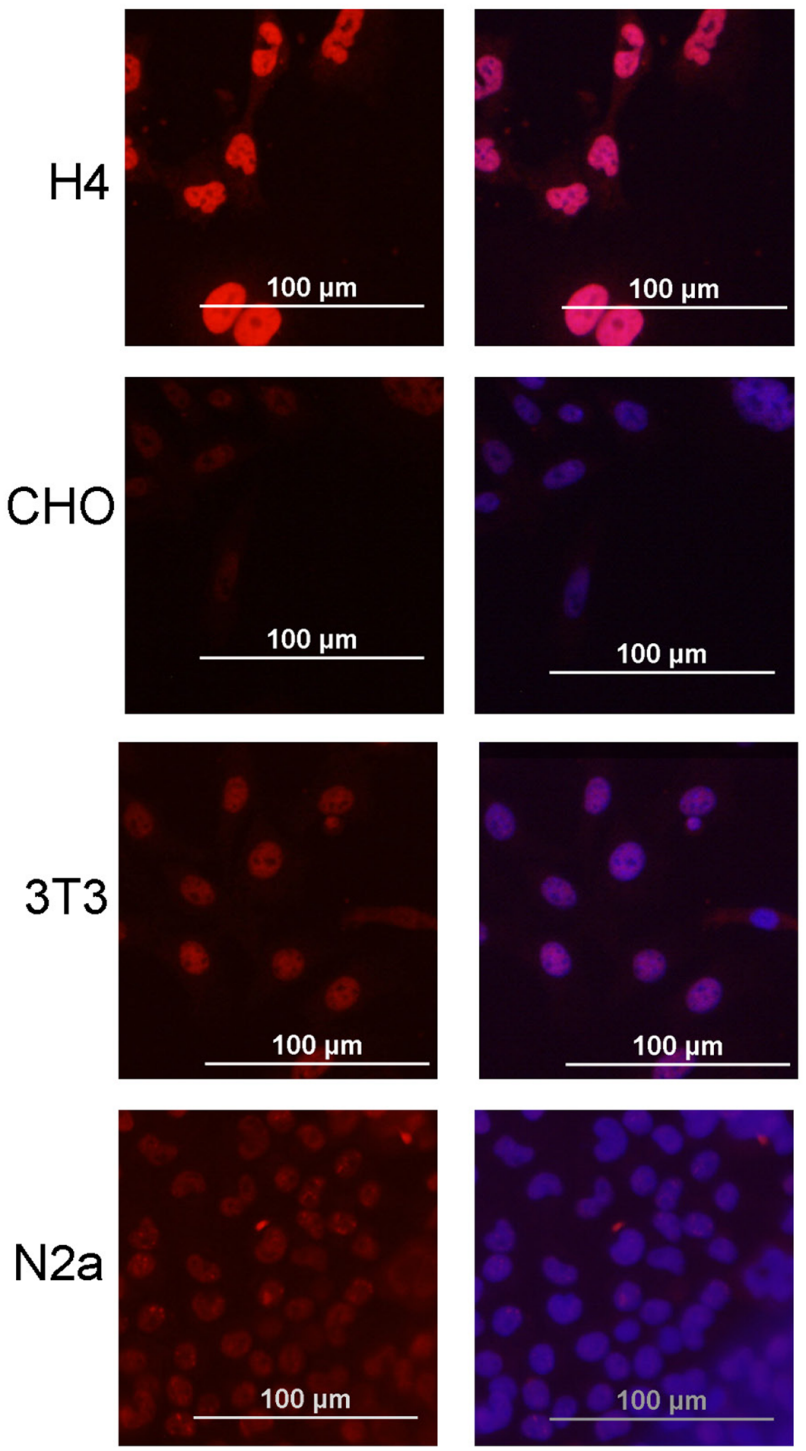
Endogenous immunostaining for Matrin 3 in untransfected cell lines. Human brain neuroglioma H4, Human Embryonic Kidney (HEK293), Chinese Hamster Ovary (CHO), Mouse Neuroblastoma (N2a), and Mouse embryonic fibroblast (3T3) were stained with α-Matrin primary antibody (ab 151714, Abcam) 1:200 overnight (Matrin 3 in red and DAPI in blue). The exposure time was set by the intensity of signal in the H4 cells. All other images were exposed to the same length of time to provide an estimation of immunostaining intensity.

Matrin 3 is a highly conserved protein, and to our knowledge there are no antibodies that can distinguish the human protein from Matrin 3 expressed by any other species. Thus, we initially screened various human and non-human cell lines to identify those with the lowest endogenous expression of Matrin 3 protein, allowing easy detection of any expressed human Matrin 3. Five cell lines commonly used in transfection studies were examined, including human H4 neuroglioma, HEK293 cells, Chinese Hamster Ovary (CHO) cells, mouse neuroblastoma N2a cells, and mouse 3T3 fibroblast cells. The weakest immunostaining with Matrin 3 antibodies was observed in CHO cells (Fig 1). By contrast, human H4 cells showed the strongest staining. In all cell lines examined, all Matrin 3 immunoreactivity was confined to the nucleus.

**Fig 2:**
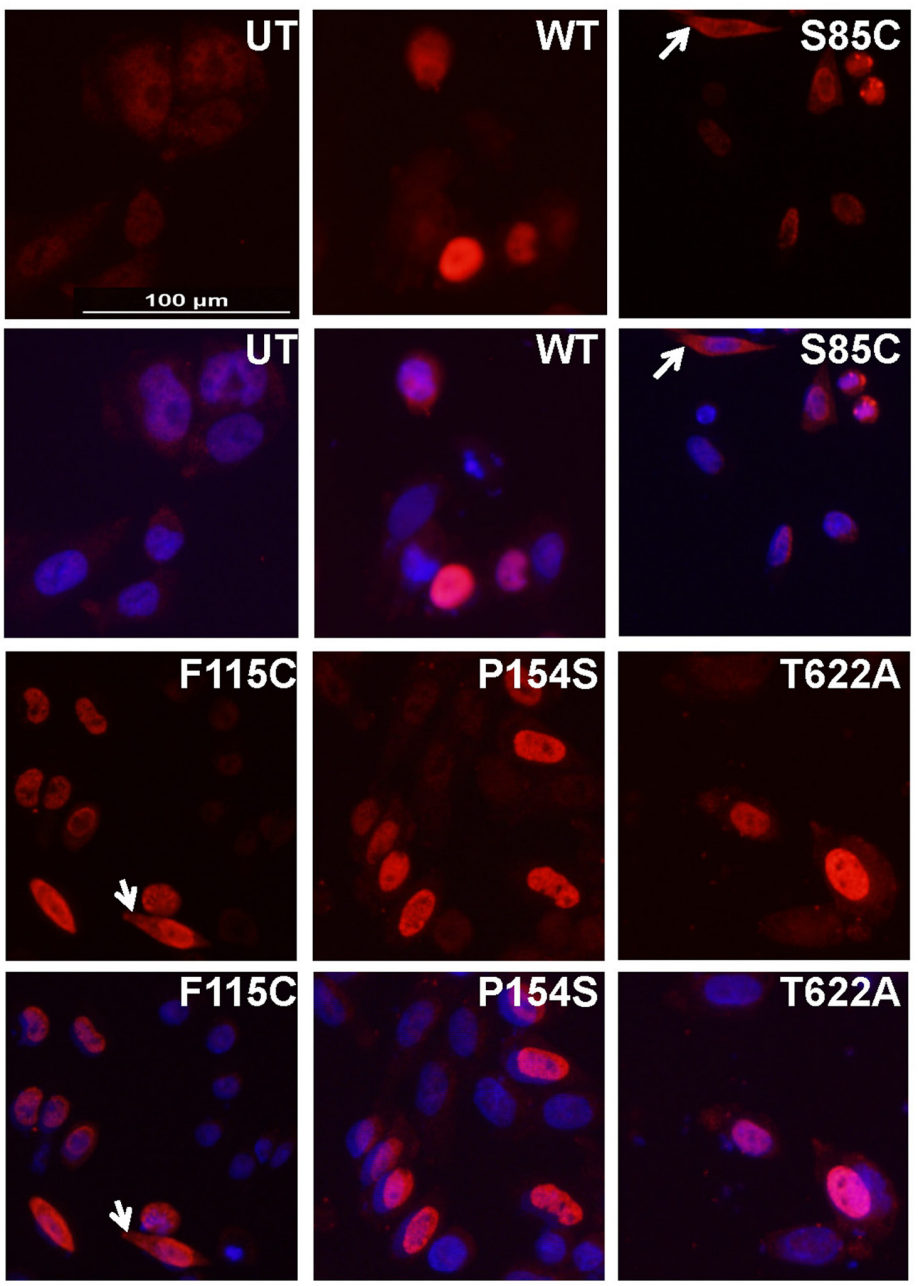
Overexpression of WT and mutant Matrin 3 in CHO cells. Cells were transiently transfected with expression plasmids for WT Matrin 3 and 4 ALS/myopathy variants; S85C, F115C, P154S, T622A. After 24 hours the cells were fixed and immunostained α-Matrin primary antibody (same as Fig 1). Most of the Matrin 3 immunoreactivity was located in the nucleus (red) but a few cells show cytoplasmic staining, particularly for the S85C and F115C variants. DAPI (blue) marks the nucleus. The exposure times for all images was identical.

Initially, based on the level of endogenous immunostaining, we chose to use CHO for transient transfection experiments to over-express wild-type (WT) human Matrin 3, and 4 different ALS/myopathy variants (S85C, F115C, P154S, and T622A). At 24 hours post-transfection, CHO cells were immunostained with anti-Matrin 3 antibody. As expected for a transient transfection, only a subset of the cells markedly over-expressed the Matrin 3 proteins (Fig 2). Regardless of whether wild-type or mutant Matrin 3 was over-expressed, the vast majority of the protein showed a nuclear localization. In cells expressing the S85C and F115C we observed some cells with obvious cytoplasmic localization (Fig 2 arrows; Table 1). However, some level of cytoplasmic staining was seen in cells expressing all variants of Matrin 3 (Table 1), and overall the groups did not differ in their frequencies of nuclear only vs nuclear/cytoplasmic localization (χ^2^ (4, N = 1179) = 3.83, p = 0.43). Consequently, the observed effect size of the association between the groups was small (ϕ = 0.06), substantiating the lack of differences between the groups. Moreover, none of the cells expressing mutant Matrin 3 were significantly different from the control, WT expressing cells, in regard to frequency of cytoplasmic staining (Matrin 3 F115C vs Matrin 3 WT, p = 0.6; Matrin 3 P154S vs Matrin 3 WT, p = 0.3; Matrin 3 T622A vs Matrin 3 WT, p = 0.6; Matrin 3 S85C vs Matrin 3 WT, p = 0.1, 2-sided Fisher Exact test). Importantly, in those cells showing Matrin 3 localization in the cytoplasm, nuclear Matrin 3 was always present and usually showed the more intense immunoreactivity (Fig 2).

**Table 1:** Localization of WT and mutant Matrin 3 in CHO cells.

Construct	Untagged Matrin 3		Matrin 3-YFP fusion proteins	
	Nuclear^#^	Nuclear and cytoplasmic*	Nuclear#	Nuclear and cytoplasmic*
**BOS-YFP**	-	-	154 (73.4%)	41 (26.6%)
**Matrin 3 F115C**	191 (65.2%)	102 (34.8%)	164 (98.8%)	2 (1.2%)
**Matrin 3 P154S**	106 (67.9%)	50 (32.1%)	218 (99.5%)	1 (0.5%)
**Matrin 3 S85C**	150 (70.8%)	62 (29.2%)	145 (95.9%)	6 (4.1%)
**Matrin 3 T622A**	162 (65.1%)	87 (34.9%)	193 (99.5%)	1 (0.5%)
**Matrin 3 WT**	169 (62.8%)	100 (37.2%)	60 (100%)	0 (0%)

^#^ Number and (percentage) of cells counted that show exclusive localization of Matrin 3 in the nucleus.

* Number and (percentage) of cells counted that show both nuclear and cytoplasmic localization.

**Fig 3:**
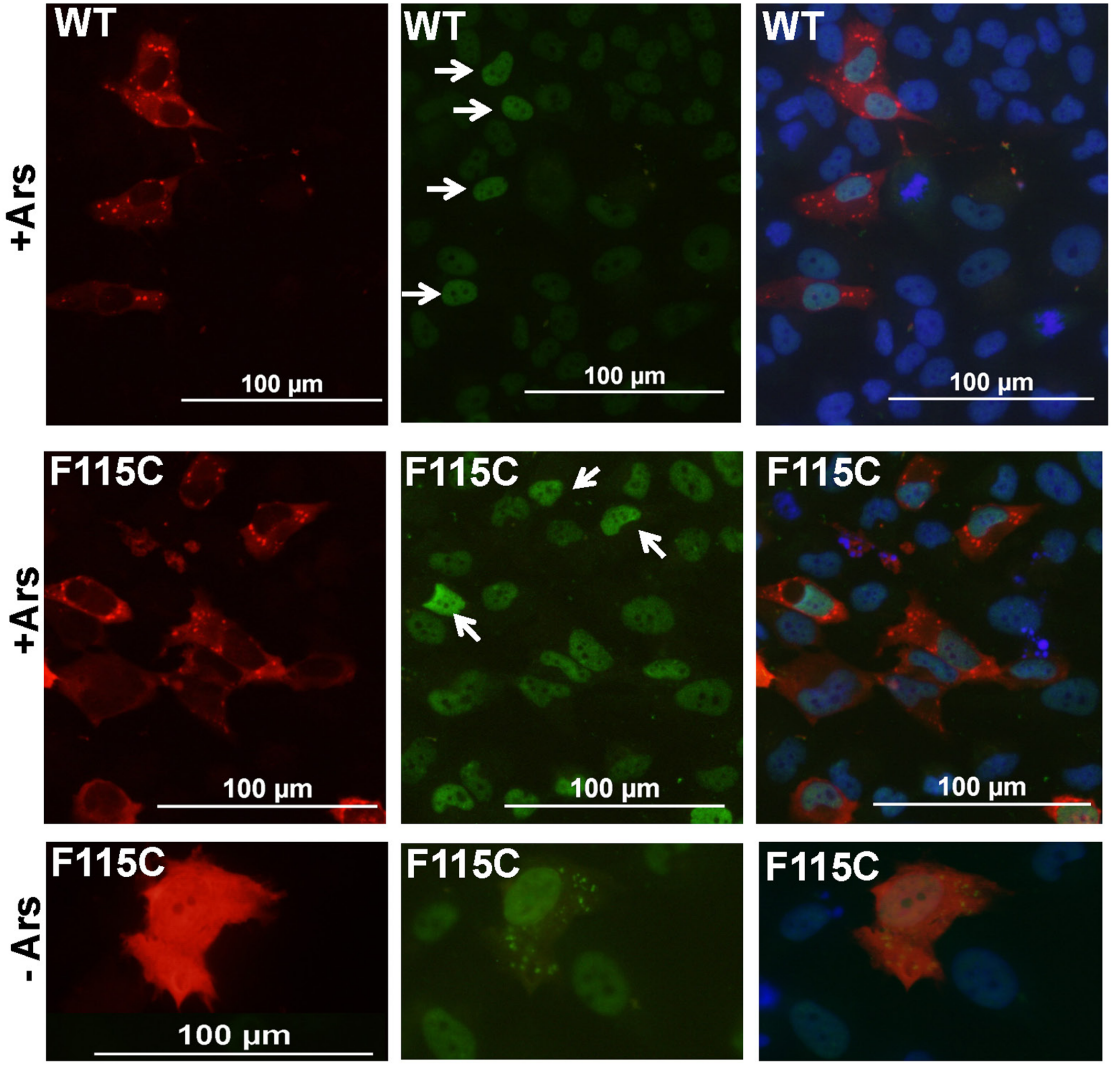
Co-expression of G3BP1-mCherry with Matrin3 in H4 cells. Cells were co-transfected with G3BP1-mCherry (RED) and WT or F115C Matrin3 and treated with arsenite (Ars) to induce stress granule formation at 24 h post-transfection. After 45 min exposure to Ars, the cells were fixed and immunostained with Matrin3 antibodies. There was no obvious co-localization of Matrin3 immunoreactivity with G3BP1-labeled stress granules. In cells that were not treated with ARS, we observed rarely cells that had Matrin3 immunoreactivity in the cytoplasm in the absence of stress granules. DAPI (blue) shows nuclear cell staining.

Previous studies have demonstrated that Matrin 3 can bind TDP-43 [3], a component of stress granules and a protein previously implicated in ALS [13,14]. To determine whether Matrin 3 may be translocated to stress granules, we used transient co-transfection in which expression plasmids for G3BP1-fused to mCherry were mixed with plasmids for our Matrin-3 constructs. Fluorescently tagged fusion proteins with G3BP1 have been widely used as a means to unequivocally identify cytoplasmic stress granules [16–18]. G3BP1-mCherry constructs have been commercialized for use as a marker for stress granules (GeneCopoeia, Rockville, MD). For these experiments, we switched to the H4 human neuroglioma cells to ensure complete species compatibility between the expressed Matrin 3 and the molecular machinery involved in stress granule formation. To induce stress granule formation, we exposed the cells to sodium arsenite (Ars) [21–23]. As expected, Ars treatment induced the formation of punctate cytoplasmic structures that have been described as stress granules [16], which contained the G3BP1-mCherry marker (Fig 3). No such structures were seen in cells co-expressing either WT or mutant (F115C) Matrin 3 with G3BP1-mCherry (Fig 3, example of cells co-transfected with F115C-Matrin 3 is shown). Immunostaining of the Ars-treated cells with Matrin 3 antibodies demonstrated nuclear Matrin 3 immunoreactivity in cells that were marked by the G3BP1-mCherry (Fig 3, arrows). In these co-transfection experiments we can have reasonable confidence that cells expressing the G3BP1-mCherry also expressed the transfected human Matrin 3 construct, and we observed modest increases in nuclear immunoreactivity in cells that expressed the stress granule marker (Fig 3). There was no obvious cytoplasmic immunoreactivity for Matrin 3 in structures labeled by the G3BP1-mCherry marker in cells co-transfected with vectors for either WT or F115C Matrin 3. An obvious limitation of this outcome is that the antibodies cannot distinguish endogenous Matrin 3 from the protein expressed by the transfected vectors. In cells co-transfected with G3BP1-mCherry and the F115C mutant Matrin 3, without exposure to Ars, we observed that a small subset of cells showed Matrin 3 immunoreactivity as cytoplasmic puncta. These puncta seemed to be induced by the co-expression of G3BP1-mCherry.

**Fig 4:**
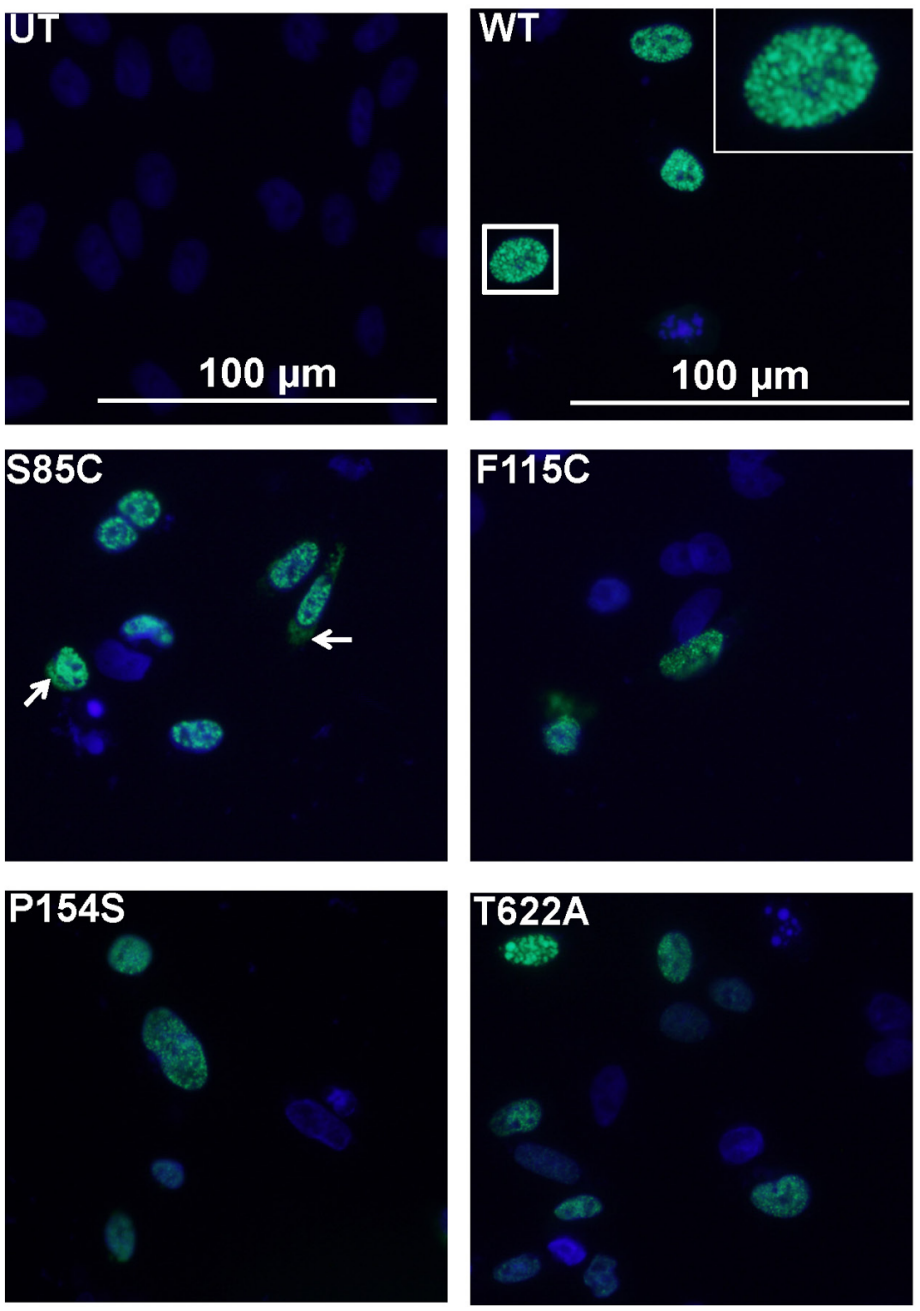
Expression of Matrin 3-YFP fusion proteins in CHO cells. Cells were transiently transfected with expression plasmids for Matrin 3-YFP fusion constructs (WT, S85C, F115C, P154S, and T622A). At 24 h post-transfection, the cells were fixed and the fluorescence was directly imaged. Nearly all of the Matrin 3-YFP fluorescence was localized to the nucleus. In a small minority of cells transfected with the S85C, we observed both nuclear and cytoplasmic localization. DAPI (blue) marks the nucleus. The exposure times for all images was identical.

To confirm our immunostaining data, we generated a panel of constructs in which YFP was fused, in-frame, to the C-terminus of Matrin 3. As above, expression plasmids for these constructs were transiently-transfected into CHO cells, but in this case we directly imaged the YFP fusion protein. As we observed with untagged Matrin 3 expressed in CHO cells, most of the Matrin 3-YFP proteins showed nuclear localization, regardless of the mutation status (Fig 4). In a small percentage of cells, particularly cells expressing the S85C and F115C variants, there was visible fluorescence in the cytoplasm (Fig 4, arrow; Table 1). In cells in which cytoplasmic localization of Matrin 3-YFP was detected, the more intense signal was always seen in the nucleus (Fig 4).

In cells in which the Matrin 3-YFP constructs were expressed, we were able to observe a little more detail on the organization of Matrin 3 in the nucleus (Fig 4, WT, inset). Most of the nuclear Matrin 3-YFP protein exhibited a punctate configuration inside the nuclear perimeter. This punctate pattern of fluorescence was seen in cells expressing both WT and mutant fusion protein (Fig 4).

**Fig 5:**
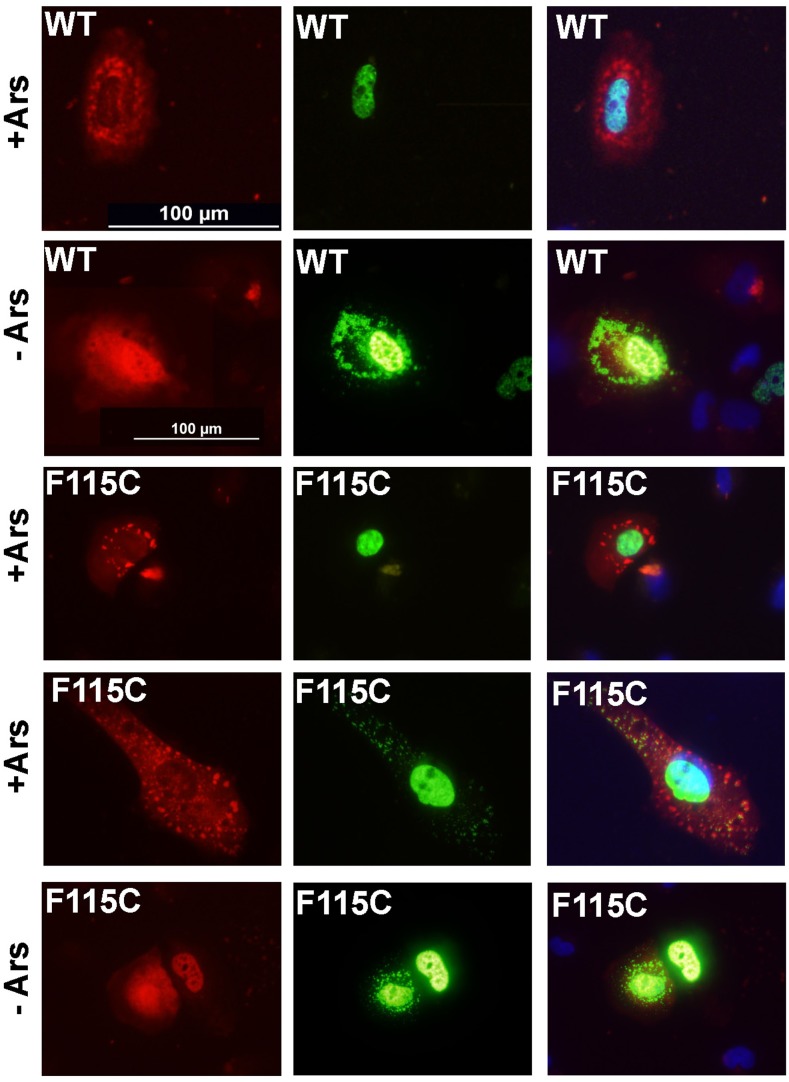
Matrin 3 is not a major component of stress granules. H4 cells were transiently co-transfected with expression plasmids for G3BP1-mCherry (red) and Matrin 3-YFP (WT and F115C). At 24 h post-transfection, one set of cells were treated with sodium arsenite [100μM] for 45 minutes to induce stress granules, which were visualized by the localization of G3BP1-mCherry. WT and F115C Matrin 3 were found to be localized to both the nucleus and cytoplasm when stress granules are present. Untreated H4 cells (-) co-expressing G3BP1-mCherry and Matrin 3-YFP also show both nuclear and cytoplasmic localization. DAPI marks the nucleus (blue). The exposure times for all images in each channel were identical; all of the images in the red channel were exposed for a shorter period than the images in the green channel.

To further investigate whether Matrin 3 may associate with stress granules, we use Ars to induce stress granule formation in cells that has been co-transfected with G3BP1-mCherry and Matrin 3-YFP. After several pilot studies (not shown), we chose conditions in which the ratio of plasmid DNA for Matrin 3-YFP to G3BP1-mCherry was set a 3 to 1, and the cells were exposed to 100 μM Ars for 45 minutes at 37°C. As was the case in the experiments described above, in all cells, the most intense fluorescence for Matrin 3-YFP was localized to the nucleus, under all conditions (Fig 5). In cells expressing the WT-Matrin 3-YFP construct that were exposed to Ars, we were able to identify clear examples in which the Matrin 3-YFP protein remained restricted to the nucleus (Fig 5). Conversely, in cells that were not exposed to Ars, but co-expressing the two fusion proteins, we occasionally observed cells in which the Matrin 3-YFP appeared to be distributed as cytoplasmic puncta (Fig 5). In cells expressing the F115C-Matrin 3-YFP mutant, we similarly observed Ars-treated cells in which mutant Matrin 3 was completely localized to the nucleus (Fig 5). There were rare examples of cells that displayed cytoplasmic F115C-Matrin 3 in structures that resembled the structures marked by G3BP1-mCherry (Fig 5). However, a pattern of similar distribution of mutant Matrin could also be found in a subset of cells that had not been exposed to Ars and had no obvious G3BP1-mCherry stress granules (Fig 5). This latter finding confirmed our observations above when untagged F115C-Matin 3 was co-expressed with G3BP1-mCherry. We conclude that co-expression of G3BP1-mCherry with Matrin 3 induces changes in Matrin 3 localization in a subset of cells, with the pattern of cytoplasmic Matrin localization resembling stress granules. Table 2 shows the results of quantification of data from multiple transfections with WT and F115C-Matrin 3 after treatment with Ars, indicating that mutant F115C-Matrin 3 is not more prone to be localized to these structures than WT protein. Based on the data in hand, we conclude that both WT and mutant Matrin 3 can form cytoplasmic structures that resemble stress granules, but there is no clear pattern of localization to indicate that the protein is actively involved in stress granule formation.

**Table 2:** Localization of WT and F115C Matrin 3 in H4 cells after stress granule induction by Ars treatment.

Construct	Untagged Matrin 3		Matrin 3-YFP fusion proteins		Total
	Nuclear + Ars^#^	Nuclear/Cytoplasmic + Ars^#^	Nuclear + Ars^#^	Nuclear/Cytoplasmic + Ars^#^	% with cytoplasmic and nuclear location
**G3BP1+Matrin 3 WT**	24	8	12	2	21%
**G3BP1+Matrin 3 F115C**	42	0	23	10	13%

^#^ Number of cells identified that display stress granules marked by G3BP1-mCherry. Columns to the right indicate the number of these cells that show nuclear localization followed by the number of cells that also showed cytoplasmic localization.

## Discussion

In this study, we determined that Matrin 3 proteins containing mutations linked to familial ALS and distal myopathy show a subcellular localization that is similar to WT Matrin 3. In multiple cell lines from multiple species (N2a, H4, CHO, and 3T3), endogenous Matrin 3 immunoreactivity was largely confined to the nucleus. In CHO cells over-expressing both wild type and disease -variant forms of Matrin 3, the protein showed primarily a nuclear localization, with limited localization to the cytoplasm. In cells expressing both WT and mutant Matrin 3 fused to YFP, we observed the fusion protein to produce a punctate pattern of fluorescence in the nucleus, suggesting binding of the protein to some substructure of the nucleus. Neither WT nor mutant Matrin 3-YFP fusion proteins seemed to be integral, consistent, components of cytoplasmic stress granules. Importantly, we did not observe that Matrin 3 harboring disease-causing mutations was prone to produce either cytoplasmic or nuclear inclusion structures that were distinctly pathologic in appearance (e.g. large perinuclear aggregates). Collectively, these data indicate that mutant Matrin 3 is not obviously distinguishable from WT protein in its subcellular localization, response to stress, or propensity to misfold and aggregate.

The data obtained by analysis of cells expressing untagged Matrin 3 was similar to that of cells expressing the YFP tagged fusion proteins in that in both cases every cell expressing the protein showed clear nuclear localization of the expressed protein. In cells expressing untagged Matrin 3, about 33% of the cells showing nuclear immunostaining for the transfected protein, both WT and mutant, also had some diffuse cytoplasmic staining (Table 1). Notably, in analyzing endogenous Matrin 3 in multiple cell lines, all visible immunostaining was nuclear. Matrin 3 has also been shown to exhibit a diffuse cytoplasmic distribution in MRC-5 cells infected with US3-negative herpes simplex virus type 1(HSV-1) or pseudorabies virus (PRV) [24]. In these virus-infected cells, phosphorylation of Matrin 3 by virus-encoded kinases, which show similar substrate preferences to protein kinase A, appeared to regulate its localization. Thus, the cytoplasmic localization in transfected cells may be due some alteration in Matrin 3 phosphorylation as a result of over-expression by transient transfection.

In cells expressing Matrin 3-YFP, in the percentage of cells showing cytoplasmic fluorescence in addition to the nuclear fluorescence drastically declined to >6% (maximum for S85C mutant, all others lower; see Table 1). The reason for this discrepancy is not entirely clear. It may be that the YFP tag on the Matrin 3 protein had some influence on the dynamics of nuclear import/export. However, arguing against this notion, we observed clear evidence of punctate cytoplasmic localization of both WT and mutant YFP fusion proteins in cells that co-expressed G3BP1-mCherry at a frequency that was no different than observed when untagged Matrin 3 was co-expressed with G3BP1-mCherry (see Table 2). At this point, we can only observe that YFP-tagged Matrin 3 proteins are primarily concentrated in the nucleus as is seen for endogenous Matrin 3.

To determine whether WT or mutant Matrin 3 may be a component of stress granules we co-expressed a well characterized marker for these granules, G3BP1 fused to a fluorescent marker (mCherry) [16–18]. Both untagged and YFP-tagged Matrin 3 proteins (WT and F115C mutant) were observed as puncta in the cytosol of cells co-expressing G3BP1-mCherry. However, these puncta were observed whether stress granules were induced by Ars or not. Although over-expression of G3BP1 has been shown to be sufficient to induce stress granule formation in the absence of exogenous stress [25], in cells expressing G3BP1-mCherry in the absence of Ars stress, all of the observable fluorescence was distributed diffusely in the cytoplasm. Thus, at present the identity of the punctate cytoplasmic structures containing Matrin 3 remains unclear. One candidate structure could be foci termed P bodies, which contain factors involved in mRNA decay (for review see [26]). However, when we attempted to detect such structures in the H4 cell model with a commercial antibody to a protein known to be located in such structures (Anti-GW182 [ab110233], Abcam)[27], we were unsuccessful (data not shown). Importantly, these cytoplasmic puncta were only observed when G3BP1-mCherry was co-expressed and thus their appearance may indicate some relationship between G3BP1 and Matrin 3 in determining the localization of the latter. However, because we observed that Matrin 3 did not translocate from the nucleus in the majority of cells that produced stress granules, we interpret our findings as evidence that Matrin 3 is not a critical component of stress granules. We also observed that a disease-causing mutation in Matrin 3 had no obvious impact on this characteristic.

Matrin 3 has been implicated in multiple cellular processes. A highly acidic domain near carboxyl terminus of Matrin, similar to High Mobility Group I/Y (HMGI and HMGY) proteins and nucleoplasmin, has been demonstrated to bind histones [8] and this acidic domain may interact with other nuclear proteins to form the internal fibrogranular network [8]. Immunostaining of cultured cells with antibodies to HMGI/Y produce a punctate nuclear distribution [28], similar to what we observed for Matrin 3-YFP. Matrin 3 has been identified in a complex that retains edited RNAs within the nucleus [29] and can regulate splicing [30]. Matrin 3 has also been identified as a component of the nuclear pore proteome [31], suggesting Matrin 3 is potentially involved in ribonucleoprotein (RNP)-complex translocation into the cytoplasm through the nuclear pore complex. The binding of Matrin 3 to unspliced and partially spliced transcripts of Human immunodeficiency virus (HIV-1) has been shown to stabilize these mRNAs and increase translocation to the cytoplasm [32]. Matrin 3 is a target for the nuclear kinase ATM, which is involved in the early stage of the double strand break (DSB) response [33]. Finally, Matrin 3 is the main substrate of PKA after activation of NMDA (N-methyl-D-aspartate) receptors [34]. PKA-mediated phosphorylation of Matrin 3 leads to its degradation in cultured cerebellar neurons, causing neuronal death [34]. Thus, Matrin 3 is implicated as a neuronal survival factor. More recently, Matrin 3 has been shown to co-immunoprecipitate with lamin-A, a nuclear matrix intermediate filament [35]; lamin-A mutations have been identified as a common cause of myopathy (for review see [36]). Collectively, these studies indicate that Matrin 3 has the capacity to modulate multiple cellular processes.

## Conclusions

Our findings in these cell culture experiments suggest that mutations in Matrin 3 that cause fALS or, in some cases distal myopathy [37], do not produce obvious changes in its subcellular localization. Additionally, we did not observe that over-expression of Matrin 3 produced the types of pathologic inclusion structures that have been observed for other proteins implicated in ALS (e.g. superoxide dismutase 1 and TDP-43). Mutations in Matrin 3 may produce disease because a function of Matrin 3 is altered. Given the multiple potential functions of Matrin 3, sorting out which function may be involved in causing ALS, or distal myopathy, will be challenging.
